# Supplementary material for: An isothermal shift assay for proteome scale drug-target identification
Source: Commun Biol. 2020 Feb 14;3:75. doi: 10.1038/s42003-020-0795-6 (PMC7021718; doi:10.1038/s42003-020-0795-6)
Supplement: Supplementary file 1 — Supplementary Information [file 42003_2020_795_MOESM1_ESM.pdf]

Supplementary Notes

TPP false negatives. As discussed in the main text, the kinobead assay identified more staurosporine targets than either the TPP or the iTSA data analysis. For the kinobead targets that could not be validated by the Savitski_TPP assay, the authors attributed the lack of significance to low replicate number and insufficient abundance or solubility resulting in lower mass spectrometry depth of coverage (MS/MS counts).[1,2] Here, we set out to evaluate some of the causes of the false negatives in the in-house TPP assay. We evaluated the thermal curves from the Ball-Webb_TPP analysis of staurosporine that validated only 28 out of the 137 detected kinobead targets. Careful evaluation of the 109 kinobead targets that could not be validated by the Ball-Webb_TPP assay indicated that small effect sizes (T_m shifts) were the greatest determining factor in whether a protein would be dismissed as a target (Supplementary Data File 1). Only 22 had a melting temperature shift of greater than 1°C. Of those, seven looked like they should be significant but lacked the statistical power to qualify as targets. Five were destabilized by staurosporine and, thus, dismissed by the one-tailed test in the TPP-TR software package. The other 10 had greater than 0.5°C standard deviation between at least one replicate.

High variance was also a likely factor in the low number of kinobead targets that could be validated with the Ball-Webb_TPP assay as, independent of effect size, 51 of these kinobead targets had greater than a 0.5°C deviation between the replicates in the Ball-Webb_TPP analysis. Lower mass spectrometry counts likely also contributed to missed kinobead target validations, with for 44 missed targets having less than 50 MS/MS counts and 14 of those having missing values in one or more replicate.

In conclusion, our analysis of TPP indicated that while mass spectrometry depth of coverage was a contribution factor to the low number of staurosporine targets validated, small effect size and high variance were more influential on statistical significance. Both of these factors could be overcome with increased sample size.

Validation of DYRK1A thermal stabilization by harmine. SK-N-BE(2) cell lysates were prepared as described in the Methods and treated with vehicle or 5 μM harmine (n=3) then processed using the iTSA workflow as described in Methods at seven temperatures: 43.9, 48, 51.2, 54.8, 57.9, 62.2°C and at room temperature. The soluble fraction was then separated by SDS-PAGE and probed by Western Blot as described in Methods using anti-DYRK1A and anti-Tubulin antibodies. As shown Supplementary Fig. 3, DYRK1A, a known binding partner of harmine, showed a significant shift in thermal stability in the presence of harmine when the melting point was evaluated by a two-tailed, homoscedastic t-test (p-value = 0.018). Likewise, a significant shift in total soluble protein was observed when the DYRK1A western was evaluated using the iso-thermal shift method near the melting point of DYRK1A (49.7°C by this detection method) (iTSA_51°C p-value = 0.042). In comparison, the loading control, tubulin, showed no significant difference in protein stability when either the melting point (61.6°C) or the iso-thermal total relative abundance was evaluated.

Supplementary References

1. Savitski, M. M. *et al.* Tracking cancer drugs in living cells by thermal profiling of the proteome. *Science* **346**, 1255784 (2014).
2. Werner, T. *et al.* High-Resolution Enabled TMT 8-plexing. *Anal. Chem.* **84**, 7188–7194 (2012).

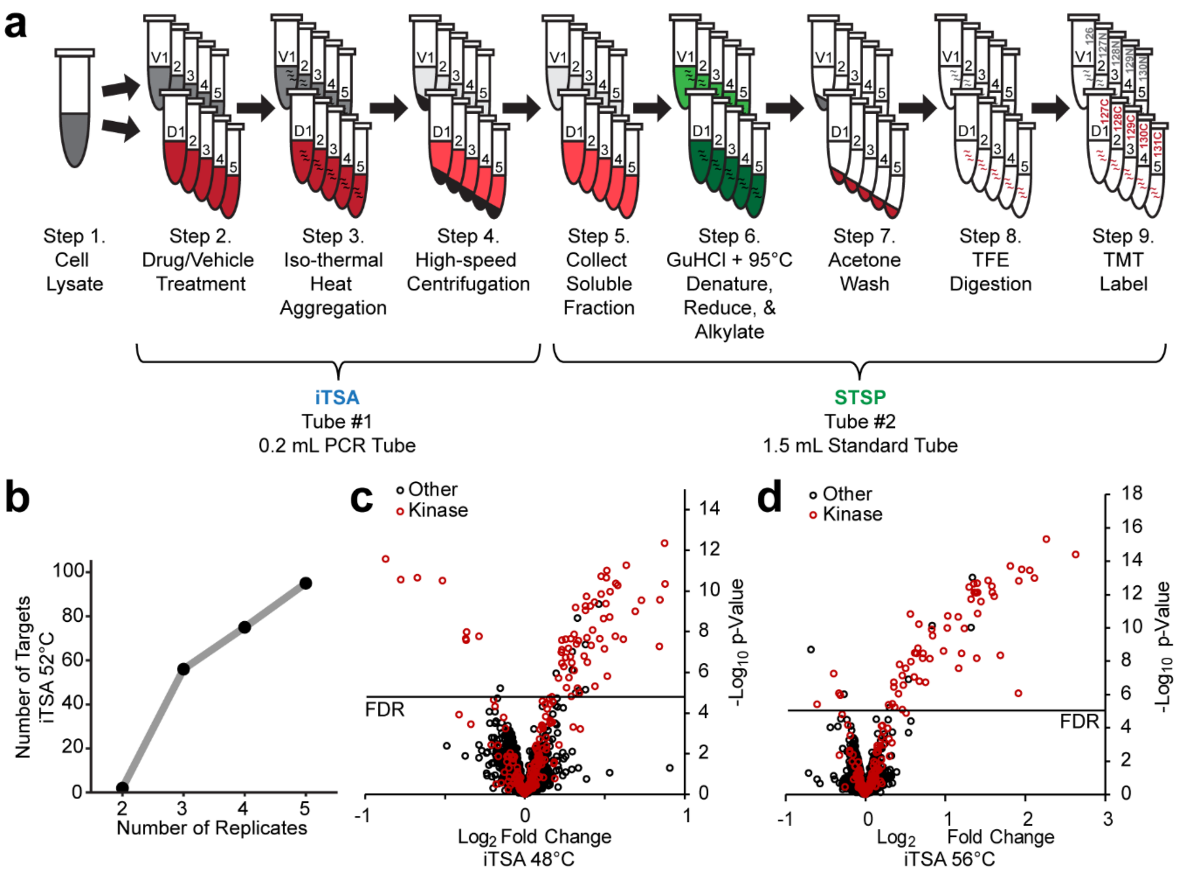

Supplementary Figure 1. **a**, Illustrated workflow for in lysate iTSA and single-tube sample prep (STSP). Procedure focuses on minimal sample handling to reduce variance between samples. Steps are described in the figure with details for each step described in Methods. **b**, Number of changes identified with increasing replication, estimated by significance (0.001 FDR) in greater than 80% of sub-sampled combinations from iTSA 52°C. iTSA was performed at 48°C **c,** and 56°C **d,** as described in Methods using 20 μM staurosporine. Volcano plot visualization of soluble protein levels using empirical Bayes statistical analysis. A positive fold change represents a more stable protein in drug condition and a negative fold change represents a less stable protein in drug condition. Proteins identified as kinases are red. FDR = 0.001 is indicated.

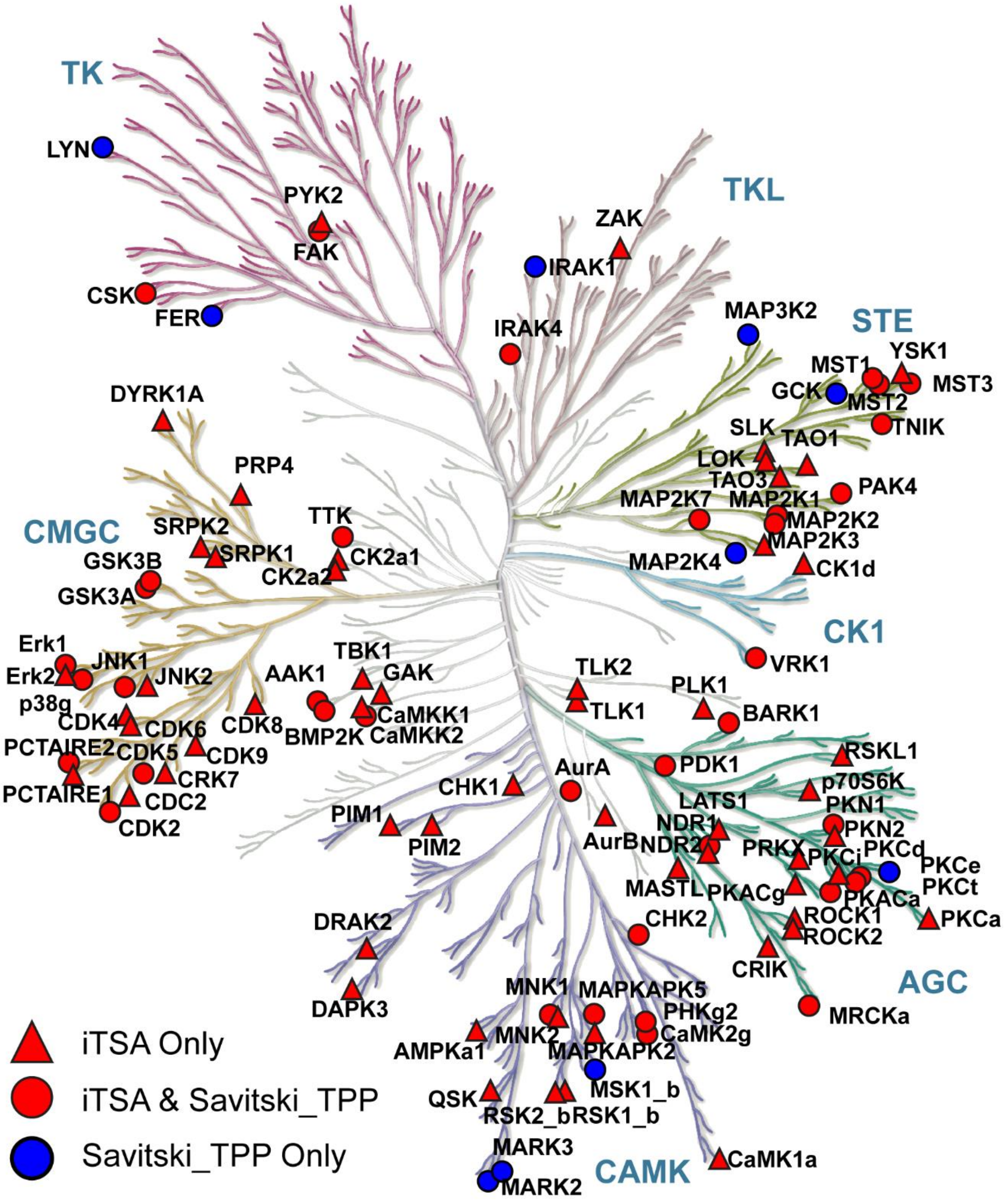

Supplementary Figure 2. Kinome map of iTSA and Savitski_TPP kinases. Blue circles represent kinases uniquely identified with TPP. Red represents kinases identified with iTSA where red triangles were uniquely identified in iTSA and red circles were targets identified by both thermal shift assays. Kinome tree illustration reproduced courtesy of Cell Signaling Technology, Inc.

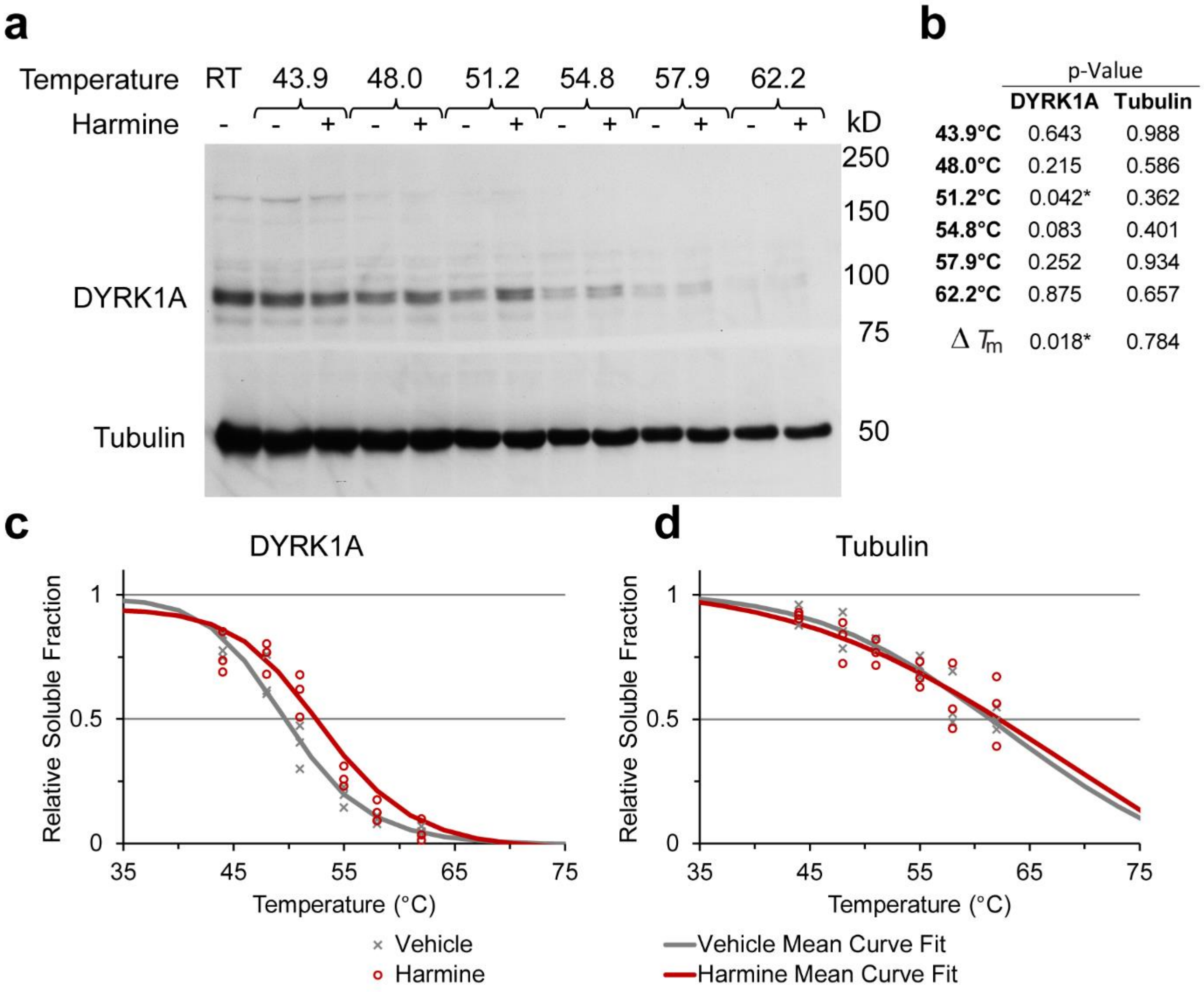

Supplementary Figure 3. Harmine dependent shift in DYRK1A protein thermal stability. The relative solubility of DYRK1A was evaluated following SK-N-BE(2) cell extract incubation with 5 μM harmine or vehicle using immunoblot quantification as described in Methods. **a**, A representative immunoblot is shown for DRYK1A and tubulin. **b**, The results of two-tailed, homoscedastic t-test (n=3) comparing vehicle and harmine is shown for each temperature and for the T_m values. T_m values determined as described in Methods. The relative quantitative data from the immunoblot analysis and curve fitting is shown for DRYK1A (**c**) and for the loading control, tubulin (**d**).

Supplementary Table 1

TMT labeling strategies											
**Experiment / TMT Label**	**1 - 126 C**	**2 - 127 N**	**3 - 127 C**	**4 - 128 N**	**5 - 128 C**	**6 - 129 N**	**7 - 129 C**	**8 - 130 N**	**9 - 130 C**	**10 - 131 N**	**11 - 131 C**
staurosporine -K562-lysate-52C-iTSA	C	D	C	D	C	D	C	D	C	D	na
staurosporine -K562-lysate-48C-iTSA	C	D	C	D	C	D	C	D	C	D	na
staurosporine -K562-lysate-56C-iTSA	C	D	C	D	C	D	C	D	C	D	na
staurosporine -K562-cell-52C-iTSA	C	D	C	D	C	D	C	D	C	D	na
staurosporine -K562-lysate-TPP	37	41.2	44	46.8	50	53.2	54	56.1	59.1	63.2	66.9
harmine-K562-lysate-52C-iTSA	C	D	C	D	C	D	C	D	C	D	D
harmine-mouse cortex-lysate-52C-iTSA	C	C	C	C	C	D	D	D	D	D	D
C = DMSO control condition; D = Drug condition; na = not applicable / no sample											
