# Supplementary material for: An isothermal shift assay for proteome scale drug-target identification
Source: Commun Biol. 2020 Feb 14;3:75. doi: 10.1038/s42003-020-0795-6 (PMC7021718; doi:10.1038/s42003-020-0795-6)
Supplement: Supplementary file 3 — Description of Additional Supplementary Items [file 42003_2020_795_MOESM3_ESM.pdf]

Description of additional supplementary items:

Supplementary Data File 1
(BallKA_WebbKJ_CommunBiol_2019_Supplementary_Data_File_1.xlsx)

This Excel workbook contains eight tables (as separate worksheets) described below.

1. StaurosporineTargetEvidence: staurosporine target identification data for iTSA analyses described in paper, merged with Savitski_TPP, Ball-Webb_TPP, and Werner_kinobead analyses.
2. Key_StaurosporineTargetEvidence: key describing the column names used in the workbook tables color coding of the column headers is consistent between key and tables.
3. boxPlot.stats: statistics of boxplots: Fig. 1b, Fig. 2e, and Fig. 2f.
4. Staurosporine_iTSA_data: mass spectrometry data and data analysis for staurosporine target identification with K562 lysates using the iTSA experimental design. This is the output files from referenced R-code with 48°C, 52°C, and 56°C output files merged together with color coding added to the column headers.
5. Staurosporine_TPP_data: mass spectrometry data and data analysis for staurosporine target identification with K562 lysates using the TPP experimental design. This is the output file from the referenced TPP package.
6. Staurosporine_Cell-iTSA_data: mass spectrometry data and data analysis for staurosporine target identification with K562 cells using the iTSA experimental design. This is the output file from referenced R-code with color coding added to the column headers.
7. Harmine_iTSA_K562_data: mass spectrometry data and data analysis for harmine target identification with K562 lysates using the iTSA experimental design. This is the output file from referenced R-code with color coding added to the column headers.
8. Harmine_iTSA_mouseCortex_data: mass spectrometry data and data analysis for harmine target identification with mouse cortex lysates using the iTSA experimental design. This is the output file from referenced R-code with color coding added to the column headers.
