# Supplementary material for: An isothermal shift assay for proteome scale drug-target identification
Source: Commun Biol. 2020 Feb 14;3:75. doi: 10.1038/s42003-020-0795-6 (PMC7021718; doi:10.1038/s42003-020-0795-6)
Supplement: Supplementary file 5 — Reporting Summary [file 42003_2020_795_MOESM5_ESM.pdf]

nature research

Corresponding author(s): William M. Old
Michael H.B. Stowell

Last updated by author(s): Dec 27, 2019

Reporting Summary

Nature Research wishes to improve the reproducibility of the work that we publish. This form provides structure for consistency and transparency in reporting. For further information on Nature Research policies, see Authors & Referees and the Editorial Policy Checklist.

Statistics

For all statistical analyses, confirm that the following items are present in the figure legend, table legend, main text, or Methods section.

n/a	Confirmed	
☐	☒	The exact sample size (*n*) for each experimental group/condition, given as a discrete number and unit of measurement
☐	☒	A statement on whether measurements were taken from distinct samples or whether the same sample was measured repeatedly
☐	☒	The statistical test(s) used AND whether they are one- or two-sided<br>*Only common tests should be described solely by name; describe more complex techniques in the Methods section.*
☒	☐	A description of all covariates tested
☐	☒	A description of any assumptions or corrections, such as tests of normality and adjustment for multiple comparisons
☐	☒	A full description of the statistical parameters including central tendency (e.g. means) or other basic estimates (e.g. regression coefficient) AND variation (e.g. standard deviation) or associated estimates of uncertainty (e.g. confidence intervals)
☐	☒	For null hypothesis testing, the test statistic (e.g. *F*, *t*, *r*) with confidence intervals, effect sizes, degrees of freedom and *P* value noted<br>*Give P values as exact values whenever suitable.*
☐	☒	For Bayesian analysis, information on the choice of priors and Markov chain Monte Carlo settings
☒	☐	For hierarchical and complex designs, identification of the appropriate level for tests and full reporting of outcomes
☐	☒	Estimates of effect sizes (e.g. Cohen's *d*, Pearson's *r*), indicating how they were calculated

Our web collection on statistics for biologists contains articles on many of the points above.

Software and code

Policy information about availability of computer code

Data collection	Mass spectrometry data collection was performed using an Orbitrap Fusion (Thermo Scientific) Xcalibur 4.0.27.42.
Data analysis	MaxQuant version 1.6.3.3, R (version 3.5.2), the R package limma and Python were used for data analysis. Details for software are provided in Materials section and a githup link is provided for access to R and Python codes. (https://github.com/CUOldLab/iTSA)

For manuscripts utilizing custom algorithms or software that are central to the research but not yet described in published literature, software must be made available to editors/reviewers. We strongly encourage code deposition in a community repository (e.g. GitHub). See the Nature Research guidelines for submitting code & software for further information.

Data

Policy information about availability of data

All manuscripts must include a data availability statement. This statement should provide the following information, where applicable:

- Accession codes, unique identifiers, or web links for publicly available datasets
- A list of figures that have associated raw data
- A description of any restrictions on data availability

Raw mass spectrometry data files (Thermo raw files) and MaxQuant output files are deposited in the MassIVE repository with the primary accession code MSV000083640. The MassIVE dataset doi is (doi:10.25345/C55036), the URI is (http://massive.ucsd.edu/ProteoSAFe/dataset.jsp?task=0171aeef20364c1e9a22501be2d8cbdf) and the ftp location is (ftp://massive.ucsd.edu/MSV000083640).

Field-specific reporting

Please select the one below that is the best fit for your research. If you are not sure, read the appropriate sections before making your selection.

☒ Life sciences ☐ Behavioural & social sciences ☐ Ecological, evolutionary & environmental sciences

For a reference copy of the document with all sections, see nature.com/documents/nr-reporting-summary-flat.pdf

Life sciences study design

All studies must disclose on these points even when the disclosure is negative.

Sample size	Five replicates were used for each condition. Using 10-plex TMT labeling, this was the maximum number of replicates that could be used with 2 conditions. Subsampling of the data is described in the manuscript and shows the limitation of using fewer samples.
Data exclusions	No samples were excluded from the statistical analysis. All outlier data points {points above and below the whiskers as defined by R boxplot()}were excluded from all box-plot figures; all of the data points are included the supplemental data files 1 and 2.
Replication	Staurosporine iTSA was performed at 3 different temperatures and was compared to previous publications. Reproducibility is discussed in the manuscript and illustrated with Venn diagrams.
Randomization	No randomization was used. Technical replicates were employed over biological replicates in this method: Drug and control samples started with a single lysate preparation that was divided into parts for 5 drug technical replicates and 5 vehicle technical replicates.
Blinding	Blinding was not performed or relevant to this method development manuscript.

Reporting for specific materials, systems and methods

We require information from authors about some types of materials, experimental systems and methods used in many studies. Here, indicate whether each material, system or method listed is relevant to your study. If you are not sure if a list item applies to your research, read the appropriate section before selecting a response.

Materials & experimental systems

n/a	Involved in the study	
☐	☒	Antibodies
☐	☒	Eukaryotic cell lines
☒	☐	Palaeontology
☒	☐	Animals and other organisms
☒	☐	Human research participants
☒	☐	Clinical data

Methods

n/a	Involved in the study	
☒	☐	ChIP-seq
☒	☐	Flow cytometry
☒	☐	MRI-based neuroimaging

Antibodies

Antibodies used	anti-DYRK1A, anti-tubulin, anti-rabbit HRP (Jackson ImmunoResearch #AB_2307391)
Validation	Anti-DYRK1A and anti-tubulin were purchased from abcam (#69811 and #18207, respectively). Please see their website for a description of the creation and validation of these antibodies (https://www.abcam.com/). The anti-rabbit HRP was purchased from Jackson ImmunoResearch (#AB_2307391). Please review their website for the validation details (https://www.jacksonimmuno.com/catalog/products/111-035-144). Antibody dilution are described in the Methods section of the manuscript.

Eukaryotic cell lines

Policy information about cell lines

Cell line source(s)	K562 (ATCC® CCL-243™) and SK-N-BE(2) (ATCC® CRL-2271™) cell lines were acquired from ATCC.
Authentication	Cell lines were not authenticated.
Mycoplasma contamination	Cell lines are tested for mycoplasma every 4-6 months using Alfa Aesar J66117 PCR Mycoplasma Detection Kit. This K562 cell line tested negative for Mycoplasma prior to and after use in this study.
Commonly misidentified lines (See ICLAC register)	*Name any commonly misidentified cell lines used in the study and provide a rationale for their use.*
